# Early and Mid-Term Outcomes of Using the Chimney Technique in Redo Mitral Valve Replacement in Patients with a Small Mitral Annulus

**DOI:** 10.3390/jcm13010270

**Published:** 2024-01-03

**Authors:** Mingyuan Yang, Laichun Song, Yong Xiao, Liang Tao

**Affiliations:** Department of Cardiac Surgery, Asia Heart Hospital of Wuhan University, Wuhan 430022, China; ymy960311@163.com (M.Y.); songlaichun@aliyun.com (L.S.); xiaoyong1431@gmail.com (Y.X.)

**Keywords:** small mitral annulus, chimney technique, redo mitral valve replacement, patient-prosthesis mismatch

## Abstract

The outcomes of redo mitral valve replacement (Re-MVR) in a small mitral annulus with the use of the chimney technique are not well documented. The purpose of this study is to present our early experience with this group of patients, illustrating the periop-erative complications and mortality outcomes. From 2019 to 2020, 77 consecutive patients underwent Re-MVR with the use of the chimney technique because of a small mitral annulus. To evaluate heart structural integrity and clinical outcomes, postoperative clinical data and echocardiograms were examined. The mean age was 56.7 ± 15.98 years. All patients underwent mitral valve surgery, of which 62 were mitral valve replacements, 7 mitral valve repairs, and 8 double valve replacements. The preoperative mitral valve mean gradient was 18.07 ± 9.40 mmHg, and the postoperative mitral prosthesis size was 28.51 ± 1.22 mm. The median increment of mitral size enlargement was 4 (0, 6) valve sizes. The mean mitral gradient coming out of the operating room was 10.34 ± 2.12 mmHg, and at the follow-up echocardiogram performed at 3 years after the procedure, it was 10.36 ± 1.70 mmHg. One-year survival was 93.3%, while the 4-year survival rate was 89.3%, with no reoperation. The use of the chimney technique in small mitral valve re-mitral valve replacement results in larger valve sizes. Moreover, the mean gradients over the mitral valve are acceptable both intraoperatively and over time.

## 1. Introduction

Performing mitral valve replacement (MVR) in patients with a small mitral annulus (<25 mm diameter) is challenging from a technical standpoint and is associated with a higher risk of unfavorable prognosis, thereby offering limited alternatives for these individuals [1,2,3,4,5]. Obviously, surgery is very difficult in patients with small mitral valve Re-MVR and involves a substantial risk of mitral annular disruption, perivalvular leak, ventricular dysfunction, and even ventricular rupture [6,7,8,9,10].

Prosthesis-patient mismatch (PPM) arises when there is a disparity between the effective orifice area (EOA) of the prosthetic valve and the necessary cardiac output to meet the demands of the patient’s body surface area (BSA) [2]. Undoubtedly, performing routine MVR in patients with a small mitral annulus without annular enlargement invariably leads to the postoperative occurrence of PPM [3]. The chimney technique was initially employed to address PPM in pediatric patients undergoing MVR [11]. The technique was subsequently reported to be used to solve PPM in adults undergoing the Bentall procedure [12] and to avoid complete mitral annular calcification (MAC) debridement in adults with severe mitral stenosis and massive MAC [13,14]. The main challenges in small mitral valve Re-MVR are the difficulty of intraoperative removal of diseased tissue from the annulus and postoperative PPM resulting from non-ideal valve size implantation. Currently, there is no feasible treatment option that can effectively address these challenges. To acquire a larger orifice area with a small mitral valve, using a regent aortic valve in mitral position is a viable option, with excellent short- and intermediate-term outcomes [5]. However, patients with small mitral annulus undergoing mitral valve replacement for the second time cannot benefit from this technique. In our institution, 77 patients with small mitral valves underwent successful Re-MVR using the chimney technique. Our intention is to present our early experience with this group of patients, illustrating the perioperative complications and mortality outcomes.

## 2. Materials and Methods

A retrospective analysis of all patients with small mitral valve who underwent a Re-MVR using the chimney technique between 2019 and 2020, with access to follow-up echocardiography, was carried out after institutional review board approval.

**Figure 1:**
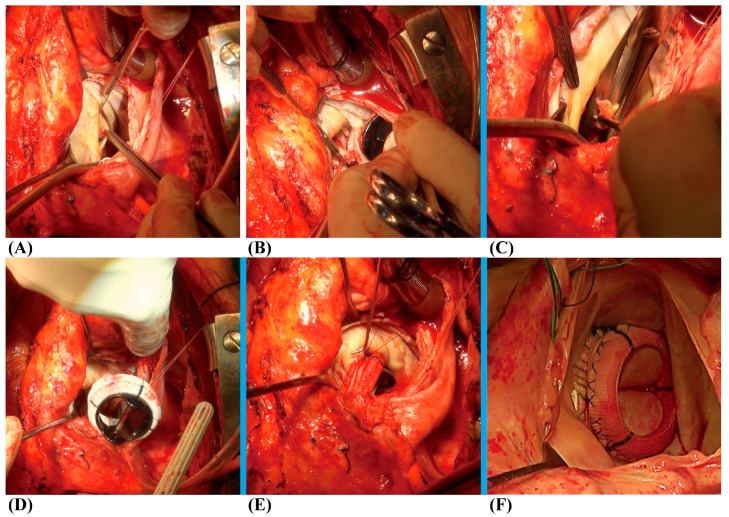
(**A**) The mitral valve was approached through an incision of the right atrium and atrial septum. (**B**) The prior mitral prosthesis was removed. (**C**) The annulus underwent significant debridement. (**D**) The valvular conduit was sutured to the annulus with a continuous stitch of 4/0Prolene. (**E**,**F**) The suture was passed from the atrial side through the left atrial outflow tract endocardium and the “chimney” of valved conduit without suturing to the prosthetic textile suturing ring.

**Figure 2:**
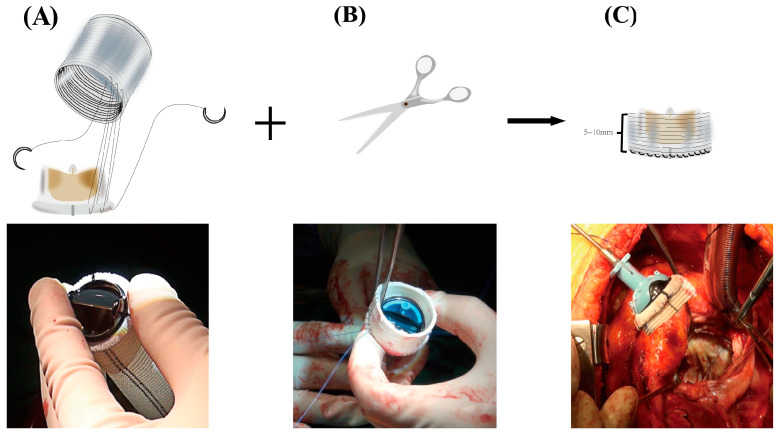
(**A**) The valve is secured within the vascular graft using running 4–0 polypropylene sutures. (**B**,**C**) The autonomously constructed valvular conduit maintains an approximate free margin of 5 to 10 mm above the valve.

Following a standard re-sternotomy, the patient receives systemic Heparin (300–400 Units/Kg). After bicaval cannulation with anterograde and retrograde cardioplegic arrest, the mitral valve was accessed through an incision of the right atrium and atrial septum. (Figure 1A). The preceding mitral prosthesis was extracted (Figure 1B), and substantial debridement was performed on the annulus (Figure 1C), leading to either complete or at least partial loss of annular tissue. The subsequent crucial phase involves the construction of a new mitral valvular conduit. A woven polyester vascular graft (InterGard; Maquet Cardiovascular, La Ciotat, France) and various valve prostheses, such as the leaflet St. Jude (St. Jude Medical, Inc., St. Paul, MN, USA) prosthetic valve, are viable options for assembling the new valvular conduit. Artificial blood vessels are at least 3 sizes larger than mechanical prostheses and 5 sizes larger than bio-prostheses. The valve is securely affixed within the vascular graft using continuous 4-0 polypropylene sutures (Figure 2A). The autonomously constructed valvular conduit maintains an approximate free margin of 5 to 10 mm above the valve. The selection of the margin’s length above the valve is determined based on the surgical requirements (Figure 2B,C). The valvular conduit was sutured to the annulus with a continuous stitch of 4/0Prolene (Figure 1D), and the suture was passed from the atrial side through the left atrial outflow tract endocardium and the “chimney” of the valved conduit without suturing to the prosthetic textile suturing ring (Figure 1E,F). Suturing should be performed with attention to the posterior annulus suture and exposure; usually, mitral valve disease is accompanied by left atrial enlargement. In most patients, the left atrium is enlarged with a septal incision, and a bi-atrial incision can be considered in patients with a small left atrium or an atrium that is not large in the first place. The cardiopulmonary bypass was ultimately weaned, and closure of the atrial septum and right atrium was performed (Supplementary Video S1). Temporary epicardial pacing wires are consistently inserted as part of standard procedure. The incision is generally closed in layers following the insertion of chest tubes and the achievement of hemostasis. Upon transfer to the intensive care unit (ICU), anticoagulation is systematically initiated at the earliest opportunity to maintain the International Normalized Ratio (INR) between 1.8 to 2.5.

### Statistical Methods

Data analysis was conducted using R software (version 4.2.1; http://www.Rproject.org; accessed on 15 July 2023). Categorical data are presented as proportions, and continuous variables are reported as mean $\bar{\chi }$ ± SD or median with date range. Estimates of survival were measured from the date of surgery to the date of death or the date of the last known time of survival (censored observations). Survival rates at 1-year and 4-year intervals were estimated using the Kaplan–Meier method. The log–log transformation method was used to calculate 95% confidence intervals (CIs) for survival. The reported statistical significance levels were all two-sided, with statistical significance set at 0.05.

## 3. Results

### 3.1. Patients’ Characteristics

The mean age of the patients was 56.7 ± 15.98 years, with 48 females and 58 patients with atrial fibrillation. All patients underwent mitral valve surgery, of which 62 had mitral valve replacements, 7 mitral valve repairs, and 8 double valve replacements. A total of 52 patients (67.5%) had undergone valve surgery 2 to 5 years prior. The mitral valve mean gradient (MG) one month after the last surgery in all patients was 11.15 ± 2.26 mmHg. The preoperative left atrial diameter (LAD) and the right ventricular systolic pressure (RVSP) were 5.14 ± 1.06 cm and 47.14 ± 9.92 mmHg, respectively. Most patients (96.1%) had underlying preoperative mitral stenosis, but 29.9% also had at least moderate mitral regurgitation preoperatively. The patients’ preoperative mitral valve mean gradient and peak gradient were 18.07 ± 9.40 mmHg and 22.43 ± 4.69 mmHg, respectively. The mean preoperative mitral valve diameter and EOAI were 24.5 ± 0.42 mm and 0.95 ± 0.27 cm^2^/m^2^, respectively. No patient had periprosthetic leakage and left ventricular outflow tract obstruction (LVOTO) (Table 1).

### 3.2. Procedural and In-Hospital Outcomes

#### 3.2.1. Procedural Data

Other concurrent procedures were performed in 42 of 77 patients (54.5%), including aortic valve replacement (*n* = 12), coronary bypass (*n* = 9), tricuspid repair (*n* = 11), modified Morrow procedure (*n* = 4), and Cox Maze IV (*n* = 12). The total cardiopulmonary bypass and aortic cross-clamp times were 179.40 ± 68.56 min and 112.90 ± 42.70 min, respectively. No intraoperative fatalities were recorded (Table 2).

#### 3.2.2. Periprocedural Outcomes

The incidence of atrial fibrillation postoperatively was 23.7% (*n* = 21). Other events and respective incidences were tracheostomy: 5.2% (*n* = 4), prolonged ventilation (>24 h): 17% (*n* = 5), reoperations due to bleeding: 3% (*n* = 1), stroke: 1.3% (*n* = 1), and temporary neurological impairment: 6.5% (*n* = 5). Mechanical circulatory support including an intra-aortic balloon was required in 1 (1.3%) patient. Two perioperative deaths occurred, both because of respiratory failure and malignant arrhythmia. The total intensive care unit (ICU) and hospital stay lengths were 6 ± 2 and 21 ± 5 days, respectively (Table 3).

**Figure 3:**
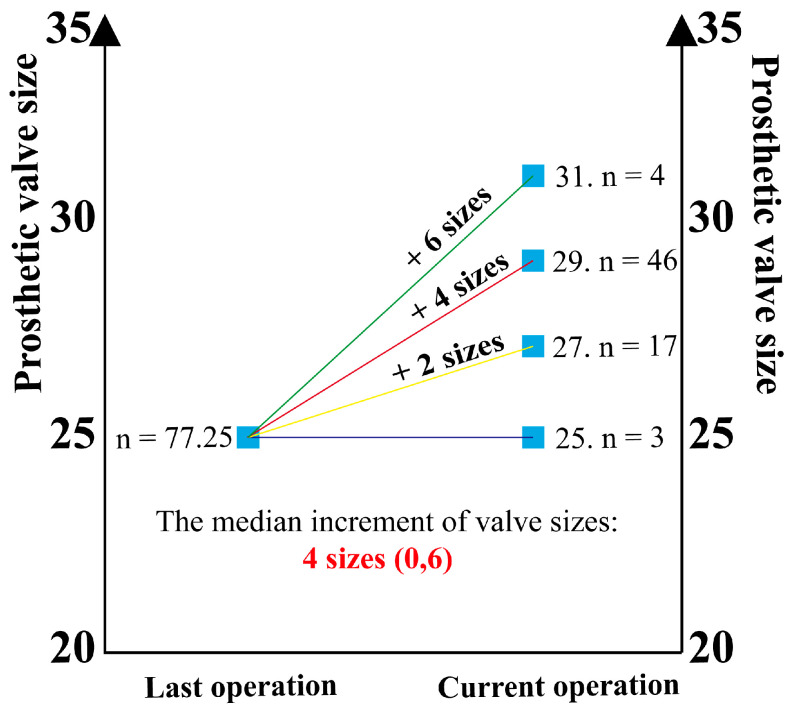
Valve prosthesis size variation (the median increment of annulus enlargement was 4 (0, 6) valve sizes).

**Figure 4:**
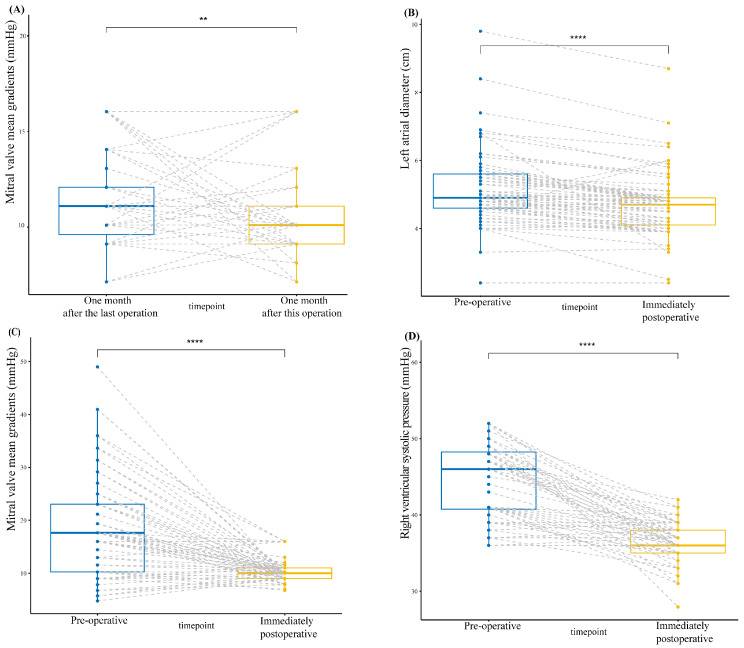
(**A**) Comparison of the MG of mitral valve prosthesis implanted one month after this operation and one month after the last operation. (**B**) Comparison of preoperative and immediately postoperative LAD. (**C**) Comparison of the MG of mitral valve prosthesis implanted preoperative and immediately postoperative. (**D**) Comparison of preoperative and immediately postoperative RVSP. ** *p* < 0.05; **** *p* < 0.001.

Postoperative transthoracic echocardiograms showed that the size of the mitral valve prosthesis was 28.51 ± 1.22 mm. Additionally, the echocardiograms also demonstrated a great performance of the composite valved conduit through the chimney technique (Supplementary Figure S1). The median increment of annulus enlargement was 4 (0, 6) valve sizes (Figure 3). The peak gradient (PG) and MG of the mitral prosthesis were 11.73 ± 3.42 mmHg and 10.34 ± 2.12 mmHg, respectively. These values were statistically significantly decreased (*p* < 0.001) compared to the preoperative data (Figure 4). The postoperative LAD and RVSP were 4.68 ± 0.92 cm and 36.30 ± 2.42 mmHg, respectively. These values were statistically significantly decreased (*p* < 0.001) compared to the preoperative data (Figure 4). The study population comprised 77.9% of patients exhibiting normal left ventricle (LV) function, 16.9% with mild dysfunction, 3.9% with moderate dysfunction, and 1.3% with severe LV dysfunction (Table 3).

### 3.3. Follow-Up Outcomes

#### 3.3.1. Clinical Outcomes

**Figure 5:**
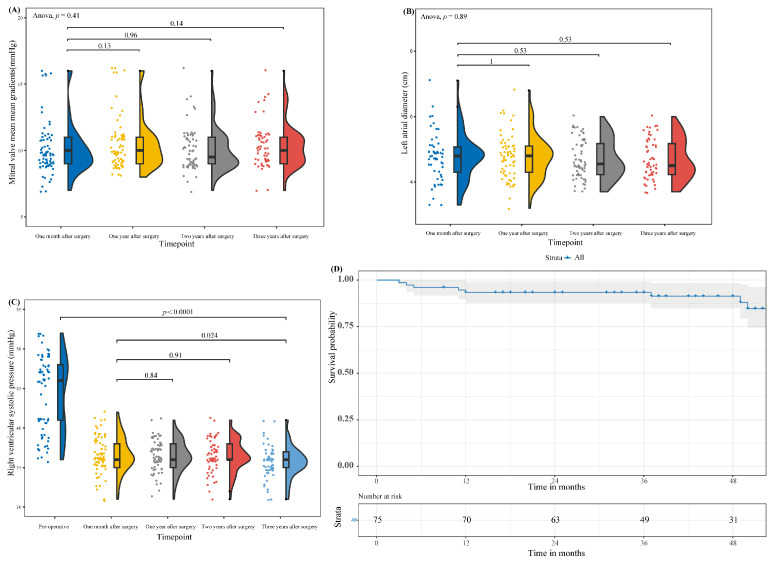
(**A**) The MG of mitral valve prosthesis implanted at 1, 2, and 3 years postoperatively compared to 1 month postoperatively. (**B**) LAD at 1, 2, and 3 years postoperatively compared to 1 month postoperatively. (**C**) RVSP at 1, 2, and 3 years postoperatively compared to 1 month postoperatively and comparison of preoperative RVSP to 3 years postoperative RVSP. (**D**) The Kaplan–Meier overall survival curve for all patients. The gray area represents the 95% confidence limits.

The mean follow-up period was 1160 ± 420 days (with 30 patients followed up beyond 4 years). Four-year patient survival was 89.3% (Figure 5). The causes of death included respiratory failure in four patients with COVID-19, sudden death in one patient, terminal stomach cancer in one patient, end-stage heart failure in one patient, and failure to thrive in a 77-year-old patient. At a median patient follow-up of 43 months (range: 3 to 54 months), no patient required reoperation and no patient had periprosthetic leakage, LVOTO, and thrombosis. The median clinical heart failure symptoms during follow-up were New York Heart Association (NYHA) class I, exhibiting a statistically significant improvement from preoperative symptoms of class II (*p* < 0.001). At the 4-year follow-up, among the surviving patients, 25 out of 30 (83%) were classified as NYHA class I, 3 out of 30 (10%) were class II (with 1 of the 3 having significant bronchiectasis), and 1 patient was classified as class III due to severe premature ventricular contractions.

#### 3.3.2. Echocardiographic Follow-Up

Follow-up echocardiography showed that the MG of the mitral prosthesis at one, two, and three years postoperatively fluctuated slightly but was not statistically significant (*p* > 0.001) compared to its measurement at one month postoperatively. Likewise, the LAD and the RVSP at one, two, and three years postoperatively fluctuated slightly but were not statistically significant (*p* > 0.001) compared to those at one month postoperatively. By the 3-year follow-up echocardiogram, the RVSP had fallen from 47.14 ± 9.92 mm Hg to 35.76 ± 2.03 mm Hg (*p* = 0.0001) (Figure 5). During the 1-month follow-up period, there was no LV dysfunction in 75% of the patients, while 21% had mild, 3% had moderate LV dysfunction, and one patient had severe LV dysfunction. During the 3-year follow-up period, there was no LV dysfunction in 91% of the patients, while 7% had mild, and one patient had severe LV dysfunction (Table 4).

## 4. Discussion

This is definitely the largest series of Re-MVR with the use of the chimney technique for a small mitral valve annulus (<25 mm). Conducting MVR in patients with a diminutive mitral annulus poses technical challenges and is correlated with an elevated risk of adverse prognosis, consequently restricting the therapeutic options for this patient subset. The chimney technique is currently described in case reports for the resolution of PPM in pediatric patients undergoing MVR. This technique enables the surgeon to install a larger valve, circumventing the necessity for re-replacement, a frequent occurrence in pediatric populations due to the growth of children [11]. Subsequently, this technique was reported to be used for PPM issues in the adult aortic valve position [12] as well as for severe MAC in MVR [13,14]. Moreover, considering that redo surgery requires extensive debridement of the mitral annulus, it may lead to two potential outcomes: firstly, excessive removal of the native valve annulus during debridement may render it impossible to suture the new prosthetic valve; secondly, the trauma from the initial surgery may result in impaired healing and scar formation in cardiac tissues, causing further narrowing of the mitral annulus, making it unsuitable for implanting an ideal size valve. The small mitral annulus may be the result of extensive calcification of the mitral annulus to the leaflets, small patient size, or previous mitral repair with a small annular ring. Surgical alternatives for Re-MVR patients with a small mitral annulus, such as extensive annular debridement and reconstruction, positioning a prosthesis in a supra-annular intra-atrial location, or deploying a transcatheter aortic prosthesis in the mitral position, are constrained and pose technical challenges [3,5,7]. Of course, there are few reports of enlarging the mitral annulus and such procedures carry a nontrivial risk of heart block. All of these techniques are associated with an increased risk of disruption of the ventriculoatrial and mitral annulus, ventricular dysfunction, perivalvular leakage, or LVOTO. In our study, the early and mid-term outcomes were outstanding, characterized by a low incidence of complications in this high-risk cohort undergoing Re-MVR.

In our series, the preoperative mitral valve size was 25#, and 96% of the patients had mitral stenosis, with a mitral EOAI of 0.95 ± 0.27 cm^2^/m^2^, all of which suggesting that all patients had severe PPM. The median increment of mitral size enlargement after MVR with the chimney technique was 4 (0, 6) valve sizes, effectively avoiding postoperative PPM. It was the enlargement of the implanted prosthesis size that significantly reduced the patients’ postoperative mitral valve MG compared to the preoperative period. Moreover, the patients’ mitral valve MG one month after this operation was 10.11 ± 1.99 mmHg, which was statistically significantly decreased from the data one month after the last operation. Thus, the chimney technique allows patients with small mitral annulus to obtain ideal valve size at Re-MVR, effectively avoiding postoperative PPM. All patients in our series had prior mitral surgery and required redo sternotomy. Additionally, the majority of patients had complex cardiac and no-cardiac comorbidities. Thus, this group of patients was a high-risk surgical group but with a relatively low postoperative complication rate.

The MG of the mitral valve after operation remained at 10.34 ± 2.12 mmHg. Additionally, follow-up echocardiographic results at one, two, and three years postoperatively showed mild stenosis of the mitral valve prosthesis (MG > 10 mmHg). This may be because the composite graft valve is not flush with the native annulus. The anterior annulus and the left atrial wall make up one side of the valve. The atrial side of the composite graft is higher than the annulus side because they are not on the same horizontal surface, which causes the valve to lean toward the annulus. The blood flowing through the valve may impact the atrial wall under the composite graft and create a turbulent flow that generates a high-pressure gradient. The postoperative high-pressure gradient of mitral valve is also partly due to the use of cardiotonic drugs. The LAD and the RVSP at one, two, and three years postoperatively fluctuated slightly but were not statistically significant (*p* > 0.001) compared to those at one month postoperatively. The LAD and RVSP follow-up results suggest that mitral stenosis is artifactual and that the mitral high-pressure gradient does not reverse conduction. The composite graft valve alters the local hemodynamics of the mitral valve, including the generation of local vortices, which may lead to the distant complication of thrombosis and pannus ingrowth in the mitral position. However, our mid-term follow-up results did not show any of these complications. In our series, the pre-existing prostheses in patients were extracted, and substantial debridement of the annulus ensued, resulting in the complete (or at least partial) excision of the annular tissue. The distal end of this valved conduit was sutured to the endocardium of the posterior wall of the left atrium, spanning from the lateral to the medial fibrous trigones. Given the fragility of the patient’s endocardial tissue, this suturing technique has the potential to result in perivalvular leakage or even avulsion of the valved conduit. However, in our cohort, periprosthetic leakage was not observed at one, two, and three years postoperatively.

The intraoperative operation of the chimney technique requires attention as follows. Firstly, there is a possibility of the valved conduit graft kinking in the left atrium due to the graft remaining free in the left atrium. The mechanical valved conduit graft height should be tailored to 5 mm, which should be sufficient to allow free movement of leaflets and to avoid kinking. However, the bioprosthetic valved conduit graft height should be tailored from 6 to 10 mm, with one side left slightly longer because the bioprosthetic valve includes the valved stent (Supplementary Figure S2). The 6 to 10 mm sleeve of the prosthetic valve rarely bends due to the use of a rigid Dacron graft. Secondly, there is a possibility that vortexing occurs locally in the mitral valve, which can lead to changes in local hemodynamics. By inclining the composite graft during trimming, so that the anterior side is higher than the posterior side, we believe that this problem can be avoided [13].

## 5. Conclusions

These results suggest that the utilization of the chimney technique in Re-MVR for a small mitral annulus presents an additional option for the replacement of small mitral valves or the achievement of annular enlargement. While circumventing the technical complexities and inherent risks associated with alternative methodologies, this approach has produced excellent short- and medium-term results in this limited series. Additional experience across multiple institutions and extensive patient cohorts will elucidate whether there are any relative contraindications to this approach or if long-term complications emerge.

## 6. Limitations

The limitation of this study is that it included a small series of highly selected patients for whom the use of the chimney technique in Re-MVR was considered to be a more feasible treatment option than alternatives such as annular enlargement and extensive annular debridement. Although there has been a short- to medium-term follow-up, there is no long-term follow-up of these patients, and exercise test results are not available for these patients. However, the improvement in clinical symptoms in the short and medium term suggests that these patients were able to benefit from this treatment in the short and medium term.

## Figures and Tables

**Table 1:** Preoperative characteristics.

Variables	*n* (%) or Mean ± SD
Demographics	
Age (years)	56.7 ± 15.98
Female	48 (62.3)
Body surface area (m^2^)	1.64 ± 0.23
NYHA III or IV	23 (29.9)
Non-cardiac comorbidities	
Hypertension	18 (23.4)
Diabetes	11 (14.3)
COPD	5 (6.5)
CKD	5 (6.5)
Stroke	10 (13.0)
Cardiac comorbidities	
Prior myocardial infarction	2 (2.6)
CAD	4 (5.2)
Atrial fibrillation	58 (75.3)
Infective endocarditis	2 (2.6)
Hypertrophic cardiomyopathy	4 (5.2)
Prior cardiac surgery	77 (100.0)
Mitral valve replacement	62 (80.5)
Mitral valve repair	7 (9.1)
DVR	8 (10.4)
One month after the last operation	
Mitral valve MG (mmHg)	11.15 ± 2.26
The last valve surgery period elapsed	
<2 years	17 (22.1)
2–5 years	52 (67.5)
>5 years	8 (10.4)
Mitral valve prosthesis	*n* = 70
St. Jude-25#	48 (62.3)
Med. Han-25#	29 (37.7)
Preoperative echocardiogram	
Mitral stenosis	74 (96.1)
Mitral regurgitation	23 (29.9)
Mitral valve MG (mmHg)	18.07 ± 9.40
Mitral valve PG (mmHg)	22.43 ± 4.69
Mitral valve annulus diameter (mm)	24.5 ± 0.42
Mitral valve EOAI (cm^2^/m^2^)	0.95 ± 0.27
LAD (cm)	5.14 ± 1.06
RVSP (mmHg)	47.14 ± 9.92
Ejection fraction (%)	52.57 ± 0.63

COPD: chronic obstructive pulmonary disease; CKD: chronic kidney disease; CAD: coronary artery disease; DVR: double valve replacement; MG: mean gradients; St. Jude: St. Jude Medical; Med. Han: Med. Hancock; PG: peak gradient; EAOI: effective orifice area index; LAD: left atrial diameter; RVSP: right ventricular systolic pressure.

**Table 2:** Operative data.

Variables	*n* (%) or Mean ± SD
Total CPB (min)	179.40 ± 68.56
Total aortic cross clamp (min)	112.90 ± 42.70
Combined cardiac procedure	
AVR	12 (15.6)
CABG	9 (11.7)
Tricuspid valve repair	11 (14.3)
Modified Morrow procedure	4 (5.2)
Cox Maze IV	21 (27.3)
Left atrial thrombectomy	3 (3.9)
Left atrial appendage closure	3 (3.9)

CABG: coronary artery bypass grafting; CPB: cardiopulmonary bypass.

**Table 3:** Postoperative complications and early outcomes.

Variables	*n* (%) or Mean ± SD
Hospital death	2 (2.6)
Reoperation for bleeding	1 (1.3)
AKI requiring dialysis	5 (6.5)
Prolonged ventilation >24 h	3 (3.9)
Stroke	1 (1.3)
Temporary neurological impairment	5 (6.5)
AF postoperatively	21 (27.3)
Tracheostomy postoperatively	4 (5.2)
IABP	1 (1.3)
Hospital length of stay (days)	20.54 ± 5.09
ICU length of stay (days)	5.78 ± 2.35
Echocardiography	
Mitral valve prosthesis size (mm)	28.51 ± 1.22
Mitral valve PG (mm Hg)	11.73 ± 3.42
Mitral valve MG (mm Hg)	10.34 ± 2.12
LAD (cm)	4.68 ± 0.92
RVSP (mmHg)	36.30 ± 2.42
LV dysfunction	
None	60 (77.9)
Mild	13 (16.9)
Moderate	3 (3.9)
Severe	1 (1.3)
Periprosthetic leakage	0
Ejection fraction (%)	49.55 ± 5.29

AKI: acute kidney injury; AF: atrial fibrillation; IABP: intra-aortic balloon pump; ICU: intensive care unit; PG: peak gradient; MG: mean gradient; LAD: left atrial diameter; RVSP: right ventricular systolic pressure; LV: left ventricular; LVOTO: left ventricular outflow tract obstruction.

**Table 4:** Follow-up.

Variables	*n*/*N* (%) or Mean ± SD			
Patients discharged	75/77 (97.4)			
Death after discharge	8/75 (10.7)			
Patients followed up at 1-year visit	69/75 (92.0)			
Patients followed up at 2-year visit	62/75 (82.7)			
Patients followed up at 3-year visit	58/75 (77.3)			
Patient follow-up beyond 4 years	30/75 (40.0)			
Mean follow-up (days)	1160 ± 420.4			
Echocardiographic data	1-month	1-year	2-year	3-year
during follow-up	follow-up	follow-up	follow-up	follow-up
Mitral valve MG (mm Hg)	10.11 ± 1.99	10.51 ± 1.97	10.03 ± 1.61	10.36 ± 1.70
LAD (cm)	4.64 ± 0.75	4.76 ± 0.67	4.70 ± 0.59	4.69 ± 0.61
RVSP (mmHg)	36.60 ± 2.25	36.48 ± 1.99	36.48 ± 2.04	35.76 ± 2.03
LV dysfunction				
None	56 (74.7)	59 (85.5)	53 (85.5)	53 (91.4)
Mild	16 (21.3)	6 (8.7)	8 (12.9)	4 (6.9)
Moderate	2 (2.7)	4 (5.8)	1 (1.6)	0
Severe	1 (1.3)	0	0	1 (1.7)
Ejection fraction (%)	51.29 ± 4.68	52.43 ± 2.99	52.56 ± 2.28	52.52 ± 2.30
Thrombosis	0	0	0	0
Periprosthetic leakage	0	0	0	0
NYHA	II	I	I	I

MG: mean gradient; LAD: left atrial diameter; RVSP: right ventricular systolic pressure; LV: left ventricle; SD: standard deviation; LVOTO: left ventricular outflow tract obstruction; NYHA: New York Heart Association.

## Data Availability

The data presented in this study are available on request from the corresponding author. The data are not publicly available due to patient privacy.

## Supplementary Materials

The following supporting information can be downloaded at: https://www.mdpi.com/article/10.3390/jcm13010270/s1, Figure S1: Postoperative echocardiography, Figure S2: Schematic diagram of the anatomical position of the chimney mitral valve, Video S1: Surgical procedure clip.

## References

[1] Urena M., Vahanian A., Brochet E., Ducrocq G., Iung B., Himbert D. (2021). Current Indications for Transcatheter Mitral Valve Replacement Using Transcatheter Aortic Valves: Valve-in-Valve, Valve-in-Ring, and Valve-in-Mitral Annulus Calcification. Circulation.

[2] Joury A., Duran A., Stewart M., Gilliland Y.E., Spindel S.M., Qamruddin S. (2022). Prosthesis-patient mismatch following aortic and mitral valves replacement—A comprehensive review. Prog. Cardiovasc. Dis..

[3] Redondo A., Lopez-Menendez J., Varela L., Muñoz R., Rodriguez-Roda J. (2018). Overcoming a Surgical Challenge: Inverted Aortic Prosthetic Valves in Small Mitral Annulus. Ann. Thorac. Surg..

[4] Cammack P.L., Edie R.N., Edmunds L.H. (1987). Bar calcification of the mitral anulus. A risk factor in mitral valve operations. J. Thorac. Cardiovasc. Surg..

[5] Barac Y.D., Zwischenberger B., Schroder J.N., Daneshmand M.A., Haney J.C., Gaca J.G., Wang A., Milano C.A., Glower D.D. (2018). Using a Regent Aortic Valve in a Small Annulus Mitral Position Is a Viable Option. Ann. Thorac. Surg..

[6] David T.E., Feindel C.M., Armstrong S., Sun Z. (1995). Reconstruction of the mitral anulus. A ten-year experience. J. Thorac. Cardiovasc. Surg..

[7] Atoui R., Lash V., Mohammadi S., Cecere R. (2009). Intra-atrial implantation of a mitral valve prosthesis in a heavily calcified mitral annulus. Eur. J. Cardiothorac. Surg..

[8] Polomsky M., Koulogiannis K.P., Kipperman R.M., Cohen B.M., Magovern C.J., Slater J.P., Xydas S., Marcoff L., Brown J.M. (2017). Mitral Valve Replacement with Sapien 3 Transcatheter Valve in Severe Mitral Annular Calcification. Ann. Thorac. Surg..

[9] Roberts W.C., Morrow A.G. (1967). Causes of early postoperative death following cardiac valve replacement. Clinico-pathologic correlations in 64 patients studied at necropsy. J. Thorac. Cardiovasc. Surg..

[10] Abramowitz Y., Jilaihawi H., Chakravarty T., Mack M.J., Makkar R.R. (2015). Mitral Annulus Calcification. J. Am. Coll. Cardiol..

[11] González Rocafort Á., Aroca Á., Polo L., Rey J., Villagrá F. (2013). Chimney technique for mitral valve replacement in children. Ann. Thorac. Surg..

[12] Song L., He G., Imin E., Tao C., Xu M., Wang B., Li X., Tao L. (2020). “Chimney” Bentall Procedure in the Small Aortic Root After Prior Aortic Valve Operations. Ann. Thorac. Surg..

[13] Go S., Furukawa T., Yamada K., Hiraoka T., Mochizuki S. (2020). A case of supra-annular mitral valve replacement using chimney technique for severe mitral stenosis with extensive mitral annular calcification. Gen. Thorac. Cardiovasc. Surg..

[14] Ma J., Tan T., Li X., Li J., Zhang Z., Yuan H. (2022). Mitral Valve Replacement Adopting Chimney Technique in Mitral Insufficiency and Extensive Mitral Annular Calcification: A Case Report. Heart Surg. Forum.

