# Supplementary figures and images for: Early and Mid-Term Outcomes of Using the Chimney Technique in Redo Mitral Valve Replacement in Patients with a Small Mitral Annulus
Source: J Clin Med. 2024 Jan 3;13(1):270. doi: 10.3390/jcm13010270 (PMC10779725; doi:10.3390/jcm13010270)

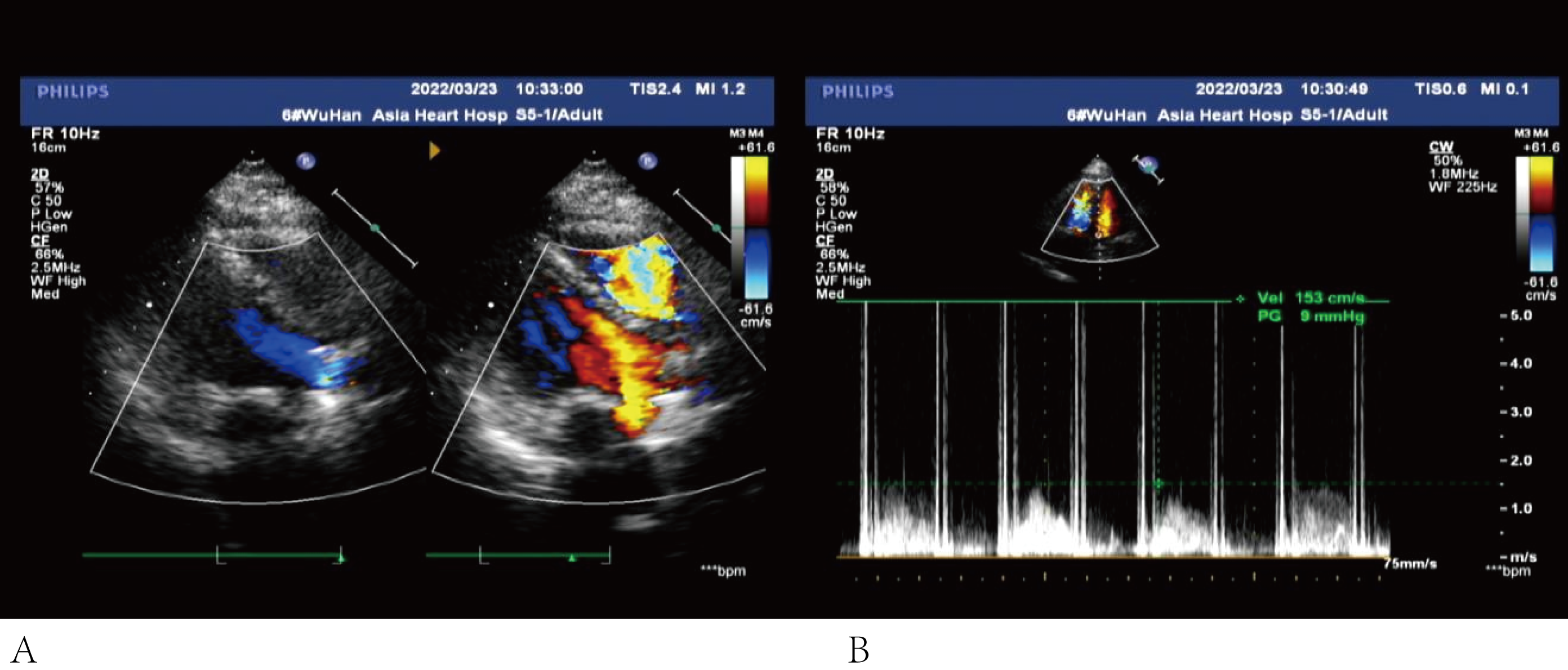

Supplement: Supplementary file 1 [file jcm-13-00270-s001.zip › Supplemental Figure S1.tif]

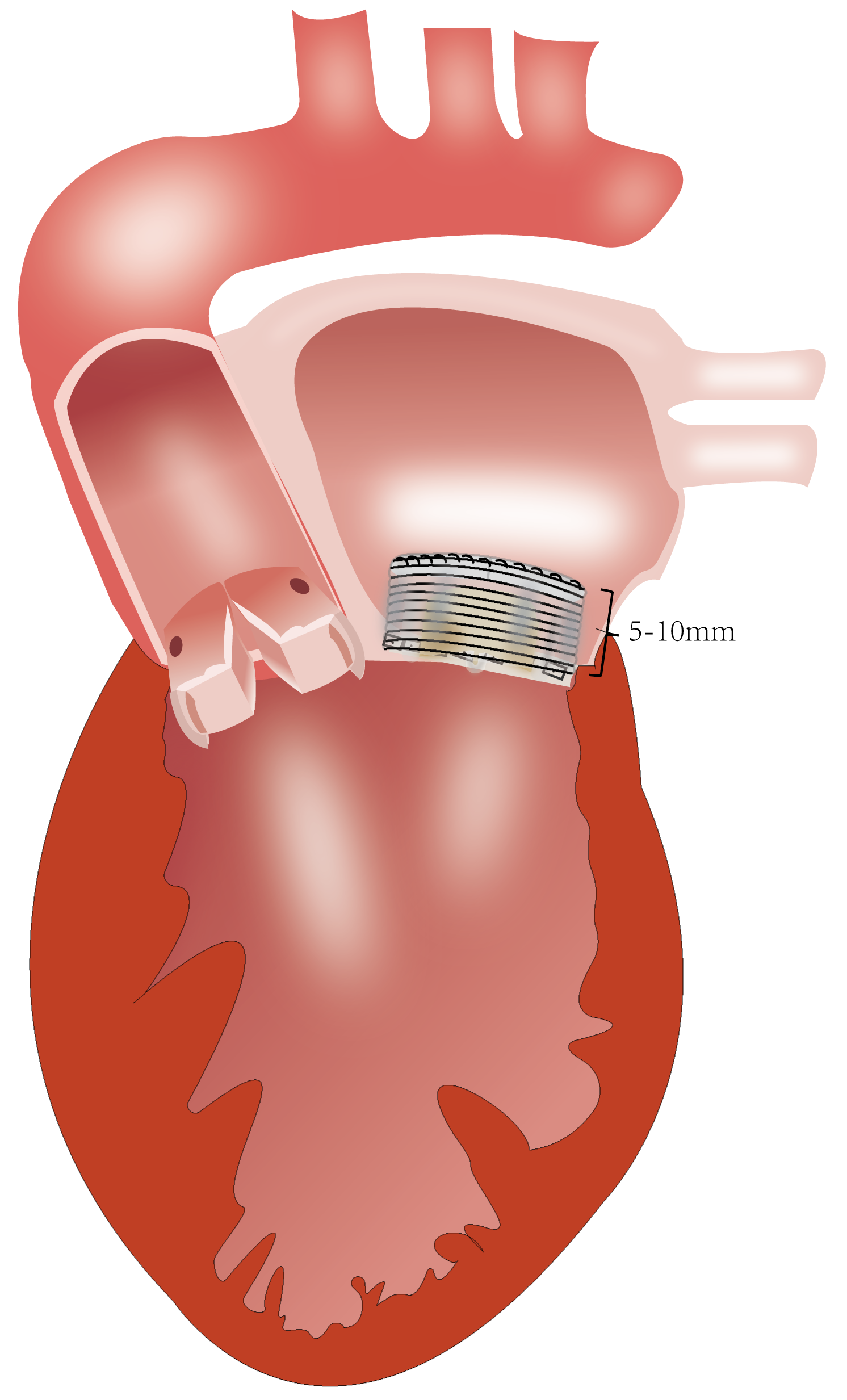

Supplement: Supplementary file 1 [file jcm-13-00270-s001.zip › Supplemental Figure S2.tif]
